# The Bull Sperm MicroRNAome and the Effect of Fescue Toxicosis on Sperm MicroRNA Expression

**DOI:** 10.1371/journal.pone.0113163

**Published:** 2014-12-02

**Authors:** Heather M. Stowe, Samantha M. Calcatera, Marcy A. Dimmick, John G. Andrae, Susan K. Duckett, Scott L. Pratt

**Affiliations:** Department of Animal and Veterinary Sciences, Clemson University, Clemson, South Carolina, United States of America; Clermont-Ferrand Univ., France

## Abstract

Tall fescue [*Schedonorus phoenix* (Scop.) Holub] accounts for nearly 16 million hectares of pasture in the Southeastern and Mid-Atlantic U.S. due to its heat, drought, and pest resistance, conferred to the plant by its symbiotic relationship with the endophyte *Neotyphodium coenophialum*. The endophyte produces ergot alkaloids that have negative effects on the growth and reproduction of animals, resulting in the syndrome known as fescue toxicosis. The objectives of our study were to identify microRNA (miRNA) present in bovine sperm and to evaluate the effects of fescue toxicosis on sperm miRNA expression. Angus bulls were assigned to treatments of either toxic or non-toxic fescue seed diets. Semen was collected and subjected to RNA isolation. Three samples from each treatment group were chosen and pooled for deep sequencing. To compare miRNA expression between treatment groups, a microarray was designed and conducted. For each of the top ten expressed miRNA, target prediction analysis was conducted using TargetScan. Gene ontology enrichment was assessed using the Database for Annotation, Visualization and Integrated Discovery. Sequencing results elucidated the presence of 1,582 unique small RNA present in sperm. Of those sequences, 382 were known *Bos taurus* miRNA, 22 were known but novel to *Bos taurus*, and 816 were predicted candidate miRNA that did not map to any currently reported miRNA. Of the sequences chosen for microarray, twenty-two showed significant differential expression between treatment groups. Gene pathways of interest included: regulation of transcription, embryonic development (including blastocyst formation), Wnt and Hedgehog signaling, oocyte meiosis, and kinase and phosphatase activity. MicroRNA present in mature sperm appears to not only be left over from spermatogenic processes, but may actually serve important regulatory roles in fertilization and early developmental processes. Further, our results indicate the possibility that environmental changes may impact the expression of specific miRNA.

## Introduction

Tall Fescue [*Schedonorus phoenix* (Scop.) Holub] is a cool-season perennial grass that is the most widely used cool-season forage in the southeastern United States. A highly adaptive grass, tall fescue possesses heat, drought, and pest resistance, which is conferred to the plant by its symbiotic relationship with the fungal endophyte *Neotyphodium coenophialum* [1], [2]. The endophyte produces ergot alkaloid compounds which are beneficial to the plant but have well established negative effects on the growth and reproduction of animals that consume the grass [3], [4]. When adjusted for inflation, the resulting syndrome, known as fescue toxicosis, is estimated to cost the ruminant production industry $1 billion annually [4]–[6].

Reproductive inefficiencies associated with fescue toxicosis have been well documented [3], with calving rates varying anywhere from 10 to 60% lower for cows consuming endophyte-infected tall fescue [7]–[11]. Research on the effects of ergot alkaloids on male reproduction, however, has been limited. Furthermore, the few studies in current literature have reported inconsistent effects of grazing infected fescue on sperm motility or morphology. Some studies indicate no effect of fescue toxicosis on sperm function [12], [13], while some report reduced sperm motility [14], [15]. Further, two studies demonstrated reduced embryo cleavage rates for sperm from bulls either given ergotamine tartrate [16] or subjected to grazing infected fescue [13], even though sperm samples showed no changes in motility or morphology between treatment groups for either study. Some of the current discrepancies in the literature may be due to confounding environmental factors, including heat stress and nutrient content of the diet, which appear to impact the extent of fescue toxicity and are difficult to control consistently [4].

One assessment not addressed in current literature is the possible effect of fescue toxicosis on sperm at a molecular level. In the last few decades, sperm have been reported to carry both RNA and microRNA to the fertilized zygote [17], [18]. MicroRNA (miRNA) are important regulators in translation, and their altered expression often leads to disease or cancer. As such, they have become popular biomarkers for diseases, cancers, or altered cellular functionality. Currently, infertility – especially male infertility – is difficult to assess and diagnosis. While the specific role of miRNA in fertility has yet to be described, miRNA has been established as important regulators of development [19]. Recent literature implicates an important role for miRNA in the post-translational regulation of spermatogenic processes (e.g., spermatogonial differentiation) [20]–[22]. Further, a recent study has demonstrated differential expression of specific miRNA between high and low fertility bull sperm [23], indicating that miRNA likely serve a role in fertility. It has also been recently shown that mature sperm deliver specific miRNA necessary for cleavage to the fertilized zygote [18]. Therefore, miRNA are logical targets for studying effects on male fertility and may provide useful biomarkers for functional, or fertile, sperm. The objectives of our study were to identify all miRNA present in bovine sperm and to evaluate the effects of fescue toxicosis on sperm miRNA expression.

## Methods

### Experimental Design

All animal research was approved by the Clemson University Institutional Animal Care and Use Committee (IACUC protocol #ARC2010-68). Angus bulls (n = 8) with a scrotal circumference (SC)>32 cm were stratified by weight, body condition score, semen quality, and SC and assigned to treatments of either toxic fescue (E+; seed containing ergovaline/ergovalanine at 2.1 mg/kg dry matter) or non-toxic fescue (E−) seed diets [24]. Bulls were provided ad libitum access to a total mixed ration containing 38% seed on a dry matter basis, formulated for 1.1 kg average daily gain and to deliver 0.8 mg/kg ergovaline/ergovalanine. Bulls were adjusted to the seed/TMR diet for two weeks prior to the study; during this time all diets contained E- seed. E+ treatment began in mid-April and continued for 126 days. Fescue toxicity in the treatment group was confirmed by monitoring levels of serum prolactin; a decrease was observed in the treatment group but not in the control group [24], a hallmark of toxicity [3].

### Sample Acquisition

Semen samples were collected at the start of test and every 28 days thereafter. To obtain concentration and motility parameters, semen samples were subjected to computer assisted semen analysis immediately following collection (data published [24]), using the SQA-Vb (Medical Electronic Systems, Los Angeles, CA) and following manufacturer’s instructions. Samples were also stained for morphological assessment using Cell-Vu pre-stained slides (Fertility Technology Resources, Marietta, GA). After collection, samples were immediately brought back to the lab for sperm cell pelleting. For pelleting, semen samples were diluted with sperm-TALP, and centrifuged to contain 3×10^7^ sperm/pellet. Pellets were flash frozen in LN_2_, and stored at −80°C. For this study, sperm pellets from the last collection (d126) were used for RNA isolation, due to treatment differences observed at that time point [24].

### RNA Isolation

Total cellular RNA, enriched for miRNA, was isolated from each sperm pellet using a modified protocol for the *mir*Vana miRNA Isolation kit (Life Technologies, Carlsbad, CA). Specifically, frozen sperm pellets were thawed on ice and re-suspended in 300 µl of solution containing 0.5% Triton and 0.1% SDS. Each sample was separated into three tubes containing 100 µl each, to which 600 µl of lysis buffer was added and vortexed well. Samples were homogenized three times with a 26 gauge needle, vortexed thoroughly, and incubated at 65°C for 30 minutes. After incubation, samples were homogenized as before with a fresh 26 gauge needle, 70 µl of homogenate additive was added, and samples were incubated on ice for ten minutes. Acid phenol:chloroform was added (700 µl) and samples were vortexed for 60 seconds prior to centrifugation at 13,000 rpm for ten minutes. In fresh tubes, one volume of chloroform was added the aqueous phase (to remove any leftover phenol contamination); samples were again vortexed 60 seconds and centrifuged at 13,000 rpm for ten minutes. Once again, the aqueous phase was placed into a fresh tube; and 1.25 volumes of room temperature 100% ethanol (EtOH) was added. Solutions were vacuum suctioned through filter columns, with the previously separated sample combined back into one sample over the filter column. Samples are separated in the beginning to provide sufficiently low sperm cell numbers to aid in cell lysis procedures, but are combined to provide adequate RNA concentration for the final resulting sample. Filter columns were washed with 700 µl wash solution 1, 500 µl wash solution 2/3, and 500 µl wash solution 2/3 (again), with 30 sec spins at 10,000 rpm between washes. After the final wash, columns were transferred to fresh tubes and centrifuged for four minutes to remove any residual EtOH. Columns were transferred to fresh tubes again and 100 µl of pre-heated (to 95°C) tris-EDTA buffer (TE) was added to each column for ten minutes and then collected via centrifugation for 13,000 rpm for one minute. The flow-through TE was re-applied to each respective column, allowed to sit for 10 minutes, and collected again at the same settings.

In order to both clean the sample of possible contaminants and to further concentrate the RNA, samples were precipitated immediately after isolation. To each sample was added: 10 µl 3.0 M NaOAc (pH 5.2), 0.67 µl glycogen blue, and 300 µl cold 100% EtOH. Once mixed together, samples were placed at −80°C overnight. For precipitation, samples were centrifuged at 17,000×g for 35 minutes at 4°C, supernatant was discarded, 1 mL cold 80% EtOH was carefully added to each pellet, and samples were centrifuged at the same settings for 5 minute; this was repeated for a second wash. For the third wash, 100% cold EtOH was used. After the third wash, supernatant was carefully removed and pellets were dried using an RNase treated spin vacuum (about 10–15 minutes). Pellets were resuspended in 50 µl (room temperature) TE.

RNA concentration and quality was determined using a Nanodrop Spectrophotometer (Thermo Scientific, Waltham, MA) and possible somatic cell contamination was determined using an Agilent 2100 Bioanalyzer (Santa Clara, CA). Samples were deemed acceptable if 260/280 ratios were greater than 1.6; 260/230 ratios were greater than 1.0; and if there was no observable somatic cell contamination (no 18S/28S peaks as determined by the Bioanalyzer). (Note: quality ratio cut-offs were recommended by LC Sciences technical staff.).

### Sequencing and Data Analysis

**Figure 1:**
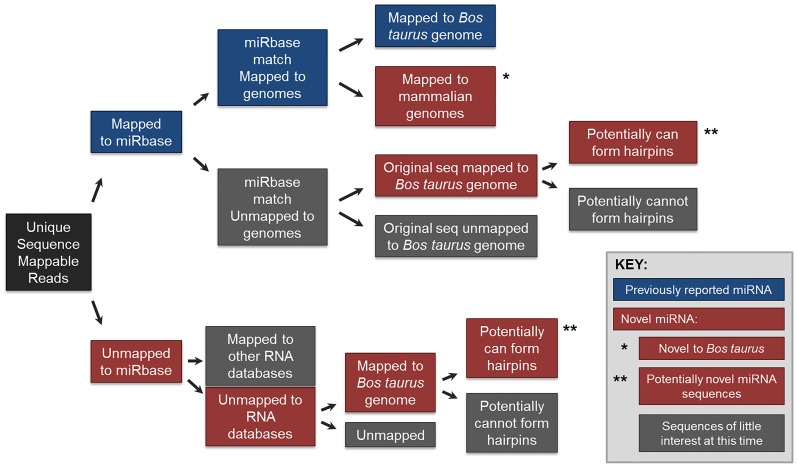
Work-flow of sequencing data analysis.

Three sperm RNA samples, from individual bulls, for each treatment group were chosen based on RNA quality (some samples did not meet quality standards as described above) and pooled for deep sequencing. For the sequencing and microarray array, LC Science’s SeqArray services were utilized (Houston, TX). Sequencing data has been submitted to Gene Expression Omnibus (accession number GSE61747). The following methods were used, as outlined by the service provider. A small RNA library was generated using the Illunima Truseq Small RNA Preparation kit according to manufacturer’s instructions. The cDNA library was used for cluster generation on Illumina’s Cluster Station and subsequently sequenced on Illumina GAIIx following vendor’s instructions. Raw sequencing reads of 40 nt were obtained using Illumina’s Sequencing Control Studio software (SCS v2.8) following real-time sequencing image analysis and base-calling by Illumina’s Real-Time Analysis (RTA v1.8.70). The extracted sequencing reads were stored and subjected to standard data analysis, utilizing a proprietary pipeline script (ACGT101-miR v4.2, LC Sciences). Briefly, unmappable reads (e.g., impure sequences) were removed and remaining sequences between 15 and 32 nt were grouped by unique sequences. Sequences were mapped against reported miRNA (miRbase version 17.0: http://www.mirbase.org/index.shtml), species’ genomes, and other RNA databases (e.g., RFam, repase, mRNA) and were classified as follows (illustrated in Figure 1):

1. Mapped to miRbase
  - Matched miRbase miRNA/pre-miRNA mapped to genomes
    - For *Bos taurus* (Group 1a)
    - For mammalian species (Group 1b)
  - Matched miRbase miRNA/pre-miRNA unmapped to genomes
    - Original sequence mapped to *Bos taurus* genome
  -
    1. Genome location potentially forms hairpins (Group 2a)
    2. Potentially cannot form hairpins (Group 2b)
  -
    - Original sequence did not map to *Bos taurus* genome (Group 3)

Did not map to miRbase

- Did not map to other RNA datebases (e.g., Rfam, repbase)
  - Mapped to *Bos taurus* genome
  1. Potentially form hairpins (Group 4a)
  2. Potentially cannot form hairpins (Group 4b)
  - Unmapped to *Bos taurus* genome (“No hit”)
- Mapped to other RNA databases

### Microarray

To compare miRNA expression between treatment groups, a microarray was custom designed and conducted by a service provider using the same three sperm RNA samples from each treatment group. The custom array was designed based on sequencing results and also included all reported *Bos taurus* miRNA. A t-test was conducted to compare normalized expression levels between treatment groups. P<0.05 was considered significant. Microarray data has been submitted to Gene Expression Omnibus (accession number GSE61747).

### Target Prediction

For each of the top ten expressed miRNA (using sequencing data), target prediction analysis was conducted using TargetScan (release 6.2; www.targetscan.org). Acquired gene names were compiled and all duplicated genes (targeted by more than one miRNA) were subjected to gene ontology (GO) enrichment analysis to determine what pathways were significantly represented by this group of miRNA. GO enrichment was assessed using the Database for Annotation, Visualization and Integrated Discovery (DAVID, v6.7, http://david.abcc.ncifcrf.gov/home.jsp). All predicted gene targets for the highest expressed miRNA (bta-miR-100) were also analyzed for GO enrichment. Only gene categories with P<0.05 were considered significantly enriched.

## Results and Discussion

### Sequencing: The Bull Sperm MicroRNAome

**Figure 2:**
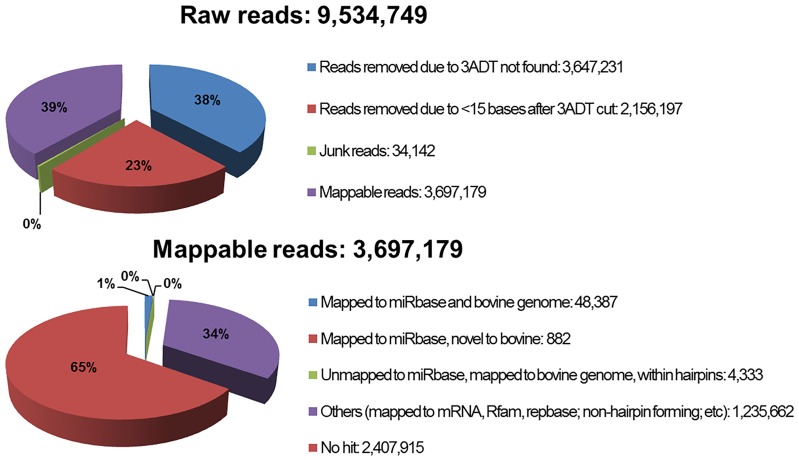
Analysis of sequencing results.

**Figure 3:**
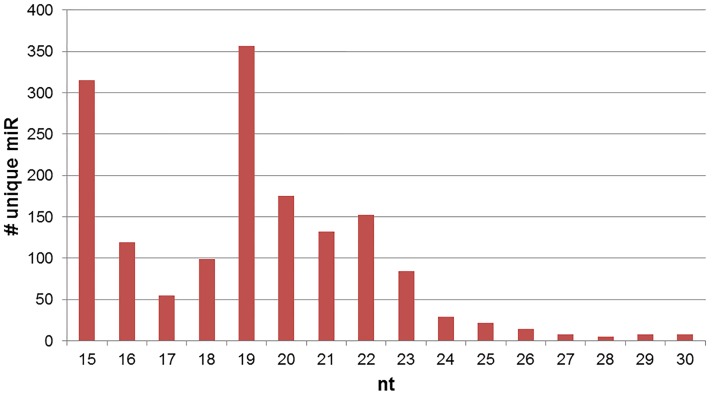
Length Distribution of Unique miRNA Sequences.

**Figure 4:**
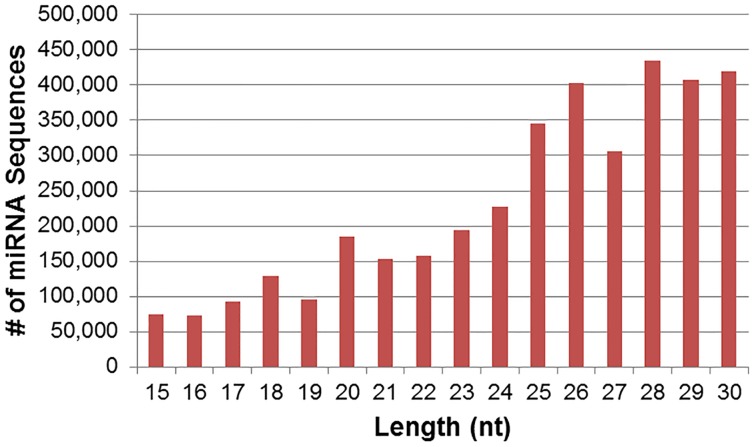
Length distribution of mappable reads.

Sequencing resulted in 9,534,749 total raw sequence reads. After filtering out impure sequences (i.e., lacking adapter sequence, junk reads, or reads <15 nt), 3,697,179 sequences remained and were considered “mappable” (Figure 2). Length distribution of unique mappable reads extended from 15–30 nt (Figure 3), with the majority of sequences at between 18 and 23 nt and a large representation at 15 nt. Notably, if graphed by the number of mappable reads (Figure 4), the majority of reads were between 25 and 30 nt. Though this appears to represent only a small percentage of unique sequences (as seen in Figure 3), this phenomenon may be due to the presence of the larger piwi-interacting RNA (piRNA) which are abundantly expressed in the testes and male germ cells [21]. This may be supported by the fact that 26.8% of the mappable sequences mapped to other types of RNA (e.g., rRNA, tRNA, snoRNA, piRNA, etc; Table 1), though the sequencing analysis did not report how many mapped to which types of RNA. Furthermore, for the purposes of this study, we were only interested in miRNA and will only further discuss miRNA sequences.

**Table 1:** Categorization of total mappable reads.

Category	# Raw Sequences	% Mappable
Group 1a	48,387	1.3
Group 1b	882	0
Group 2a	1,015	0
Group 2b	1,576	0
Group 3a	1,566	0
Group 3b	595	0
Group 4a	4,333	0.1
Group 4b	146,381	4
Mapped to mRNA	183,069	5
Mapped to other RNAs	991,929	26.8
Mapped to Repbase	109,696	3
Nohit	2,407,915	65.1
**TOTAL MAPPABLE READS**	**3,697,179**	**100**

Mappable sequences were grouped according to several criteria (as outlined previously; results depicted in Table 2). In total, 1,582 unique miRNA were identified. Of those sequences, 382 were known *Bos taurus* miRNA (Group 1a), 22 were known but novel to *Bos taurus* (Group 1b), and 816 were predicted candidate (PC) miRNA that did not map to any currently reported miRNA (Group 4a). The other miRNA sequence categories (Groups 2 and 3) mapped a total of 641 miRNA; however, with the exception of Group 2a, these sequences may be of little interest given their inability to map to genomes of interest (neither specifically *Bos taurus* nor generally mammalian species). Group 2a, which totaled 8 miRNA, may be of some interest as the original sequence read did map to the *Bos taurus* genome and these genome locations could potentially form hairpins. In this case, these are previously unidentified miRNA in *Bos taurus*.

**Table 2:** Known and predicted miRNA detected in bull sperm.

		Group	#UniquemiRNA
**Known miRNA**	Of specific species^1^	1a	382
	Of selected species^2^, but novel to specific species	1b	22
**Predicted miRNA**	Mapped to known pre-miRNA of selected speciesand genome; within hairpins	2a	8
	Mapped to known pre-miRNA of selected speciesand genome; no hairpins	2b	202
	Mapped to known pre-miRNA and miRNA of selectedspecies but unmapped to genome	3a	383
	Mapped to known pre-miRNA of selectedspecies but unmapped to genome	3b	48
	Unmapped to known miRNA but mapped to genomeand within hairpins	4a	816
	**Overall (Unique miRs)**		1582

1 Bos taurus.

2 Mammalia.

Of the miRNA detected in bull sperm, only 256 exhibited a copy number greater than 5. Thirty-five miRNA exhibited a copy number greater than 100 (Table 3). Of those, four miRNA had a copy number greater than 1000 (miR-10a, −10b, −34c, and −100) and one had a copy number greater than 10,000 (miR-100). Several of the top 35 miRNA actually turned out to not be miRNA at all. A miRBase inquiry of each miRNA revealed that four of the reported miRNA sequences have been retracted as miRNA when their sequences were determined to be pieces of either tRNA or rRNA. These include: mmu-mir-5102-p3, ssc-miR-2476, and sha-mir-5105-p3 (note that ssc-miR-2476 is included twice in the top 35, with two different isomiRs: ssc-miR-2476_R+5 and ssc-miR-2476_R+4; the sequence is noted to be a part of a tRNA).

**Table 3:** MicroRNA sequenced in bull sperm with greater than 100 copy number; previously reported sequences have been noted.

No.	miRNA Name	nt	Copy #	Group	Boarsperm[45]	Bullsperm [23]	Humansperm [46]
1	bta-miR-100_R-1	21	18154	1a	+	+	+
2	bta-miR-34c_R+1	23	1939	1a			+
3	bta-miR-10b_R-1	22	1888	1a	+		
4	bta-miR-10a_R-1	22	1206	1a	+		+
5	bta-miR-99a	21	832	1a	+		+
6	bta-miR-27b	21	661	1a	+		+
7	bta-miR-204	22	493	1a	+		
8	bta-miR-26a	22	457	1a	+		
9	bta-miR-146a_R-2	22	394	1a	+		
10	bta-miR-191_R-3	20	348	1a	+	+	+
11	bta-miR-186_R-1	21	307	1a	+		
12	bta-miR-21_R-2	22	287	1a			+
13	bta-miR-2284x_R+1	22	264	1a			
14	bta-miR-30d_R-4	20	255	1a	+		
15	bta-miR-143_R-2	20	255	1a			
16	bta-miR-335	23	234	1a	+		+
17	ssc-miR-2476_R+5	26	226	2b			
18	eca-miR-34b-3p_2ss9AG10CT	22	209	1a	+		+
19	bta-miR-199a-5p_R+1	23	209	1a			
20	sha-mir-5105-p3_2ss5AG22AG	24	199	2b			
21	bta-miR-151*_R-1	20	198	1a	+		
22	bta-miR-22-3p_R-2	19	181	1a	+		+
23	PC-3p-3747_181	18	181	4a			
24	bta-let-7a	22	156	1a	+		
25	bta-miR-24-3p_R-2	20	155	1a	+		
26	bta-miR-125b	22	153	1a	+	+	
27	ssc-miR-2476_R+4	25	138	2b			
28	bta-miR-30e-5p	24	137	1a	+		+
29	hsa-miR-1290_1ss13TG	19	130	2a			
30	mmu-mir-5102-p3	25	116	2b			
31	bta-miR-148a	22	111	1a	+		+
32	bta-miR-16b_R+1	22	109	1a	+		
33	bta-miR-16a_1ss21TC	22	109	1a	+		
34	bta-let-7b	22	106	1a	+		+
35	bta-let-7i	22	103	1a	+		

+ miRNA have been previous reported.

It is also interesting to note that many of the miRNA sequences obtained were not identical to the reported miRBase sequence for the reported miRNA. This is the phenomenon known as isomiRs, in which the mature miRNA sequence may vary by one or more nucleotides. Such changes may involve substitutions, insertions, or deletions in the miRNA sequence (less common), or cleavage-variations whereby the isomiR sequence is either shorter or longer than the canonical miRNA on either end, 5′ or 3′ (more common). Cleavage-variations are due to differences in processing by either Drosha or Dicer [25], with 3′ end variations observed much more often than 5′ [26]. Though they were originally thought to be random occurrences, many studies are describing non-random patterns of isomiR expression, implicating functional roles for isomiR distribution (though specific functions are as of yet unknown) [26]–[28]. Interestingly, the “dominant” isomiR may vary according to species and tissue type, and may be different than the reported miRBase sequence [25], [26]. In all tables reporting miRNA in this manuscript, we note by name specifically which miRNA have altered sequences. For example, miR-100_R-1 indicates a subtraction of one nucleotide from the right side. However, for ease of reading the text, we refer to the miRNA by its base name only (i.e., miR-100).

Several of the top expressed miRNA in this study have been previously reported in sperm from other species (Table 3). Several of these were found to be differentially expressed in abnormal versus normal human sperm, including: miR-26a, −99a, −16, and −34b-3p [29]. Differential expression of miRNA between normal and abnormal sperm has also been demonstrated in pigs, with let-7a and miR-22 showing increased expression in sperm with abnormal morphology [30]. These miRNA may be useful indicators of fertility potential/biomarkers for fertility.

Sperm miRNA expression between bulls of low and high fertility was recently evaluated using microarray [23]. Several of the reported top 10 miRNA expressed in both high and low fertility bulls were consistent with our sequencing results (miR-191, −125b, and −100) while others were not (miR-638 and −289). (Note: the “top 10″ were reported as species specific miRNA, and therefore several were duplicated on the list, e.g., hsa-miR-100 and dme-miR-100.) Many of the top 10 miRNA were consistent in both high and low fertility bull sperm. There were several miRNA with differential expression between bulls of high and low fertility, however these miRNA were novel miRNA reported as “hsa-asg-#.” Nevertheless, some of these sequences did match sequences obtained in our study. The most notable example being hsa-asg-14189, whose sequence matched to ssc-miR-1285-p5 in our probe set. Important biological and technical discrepancies exist between our study and the fertility based study, however, which could account for the differences in observed miRNA expression. For instance, mature progeny tested Holstein bulls were utilized in the fertility study, versus the younger Angus bulls in this study. Further, the fertility study utilized a discovery-oriented microarray, which omitted some relevant species (e.g., sus scrofa). Our results, on the other hand, accounted for sequencing of all present sequences, which were then mapped to all reported miRNA sequences.

The majority of literature concerning specific miRNA physiology focuses on the role of miRNA in cancer pathways, though a few studies have begun to determine the role of specific miRNA in germ cell development and early embryonic pathways. For instance, the let-7 family and miRNA-146 regulate spermatogonia differentiation [31], [32], though through different mechanisms. Both are affected by retinoic acid, though let-7 is up-regulated [32] and miRNA-146 is inhibited by retinoic acid [31]. Let-7 appears to induce differentiation pathways through the inhibition of Lin28, among several other targets [32]. MiRNA-21 has a role in maintaining spermatogonia stem cells by regulating apoptotic pathways [33]. Of the fifteen top expressed miRNA in undifferentiated spermatogonia (*in vitro*), six were present in the mature sperm we analyzed, including: let-7a, −7b, miR-26a, −16, −125b, and −24 [20]. MiRNA-16 is known to regulate cell-cycle progression [34]. The presence of these miRNA in mature sperm may simply be a leftover product of the earlier spermatogenesis pathways. However, the fact that some miRNA from early spermatogenic processes are present in mature sperm while others are not may indicate other roles for these miRNA either later in sperm development or in subsequent processes such as fertilization.

While many of the top expressed miRNA in mature sperm may seem to be remnants of spermatogenesis, others may have functionality in downstream processes and have been reportedly involved in early embryonic development and other developmental processes. Seven miRNA (five of which were present in our study: let-7a, −7b, miR-125b, −10b, and −26a) have shown differential expression during quail embryonic development and are postulated to function in quail embryo somite development [35]. Let-7a has also been shown to have an inhibitory effect on blastocyst implantation in mice by targeting integrin-β3 [36]. Among the 21 differentially expressed miRNA between undifferentiated mouse embryonic stem cells (ESC) and differentiated embryoid bodies (EB), five were present in our study: let-7b, miR-335, −125b, −100, and −2 [37]. Of these, all except miR-335, were more highly expressed in ESC and showed down-regulation in day 3 and day 7 EB. The study goes on to further investigate the role of miR-125b, demonstrating that miR-125b down-regulates endodermal and mesodermal formation and suppresses cardiomyocyte differentiation by targeting Lin28 [37]. MiR-125b also regulates osteoblast differentiation from mesenchymal stem cells, having higher expression in undifferentiated cells [38]. Notably, miR-125b/let-7a/miR-100 (all highly expressed in our study) are clustered together in the human genome [39]. Another miRNA which was expressed in the mature sperm in this study, miR-335, is also expressed in oocytes, but shows reduced expression in embryos; altered expression of this miRNA affects spindle formation and polar body formation, though it does not appear to affect subsequent embryonic development [40]. Given the effect window for miR-335 in oocyte development but lack of embryonic affect, sperm contribution of this miRNA may have little consequence on subsequent developmental processes. All of these miRNA have widely varied expression patterns, timing, and affects in developmental processes, demonstrating the intricate and complicated nature of miRNA regulatory mechanisms. It is difficult to postulate at this time whether sperm contribution of these miRNA to the fertilized zygote affects downstream developmental processes; however, the complex nature of these pathways along with currently reported miRNA involvement in development makes the proposition an intriguing, and perhaps likely, one.

### MicroArray Results: Effect of Fescue Toxicosis on Bull Sperm miRNA Expression

A total of 2,931 miRNA sequences were probed for, based on sequencing results and including all reported bovine miRNA. Of the sequences chosen for microarray, 22 showed significant differential expression between treatment groups (P<0.05; Table 4), 11 of which were at low signals (detected signal intensity <500, based on a range from 1 to over 65,000). A total of eight miRNA were down-regulated (4 at low signals) and 14 were up-regulated (7 at low signals) in the treatment group as compared to control. Of the 22 differentially expressed miRNA, 17 were from Group 4b – the group of sequences that did not map to miRBase, did map to the *Bos taurus* genome, but whose sequences cannot potentially form hairpins. Given that miRNA precursors are known to form hairpins, these “miRNA” sequences are likely not actual miRNA. Given the number of probes included in the microarray, it is possible that some of these differentially expressed sequences are false positives. None of the differentially expressed sequences in this study matched to the differentially expressed miRNA sequences reported between high and low fertility bulls [23]. As previously mentioned, this could be due to a number of biological factors (e.g., breed and environmental differences), as well as technical factors (i.e., procedural differences). Of the remaining differentially expressed sequences in our study, only two (miR-146a and −146b) are sequences that are previously reported miRNA sequences, one of which was not detected in the sequencing results (miR-146b).

**Table 4:** Differentially expressed miRNA in bulls affected by fescue toxicosis.

			Control		Treatment		
miRNA Reporter Name	Group	nt	Mean	StDev	Mean	StDev	Target Sequence (5′ to 3′)
PC-3p-37804_13	4b	20	670	124	1,369	267	AAGAACUUUGAAGAGAGAGU
PC-5p-143070_3	4b	26	298	52	556	105	GUUGUGGUAUAGUGGUUAGCAUAGCU
PC-3p-8404_71	4b	20	779	564	91	61	AAAAUUUGGAGAGUUUGAUC
PC-5p-9265_64	4b	18	6,182	4,429	211	305	AUUACCUGCUGUUCGAUU
PC-3p-172110_3	4b	19	22,509	8,014	54,331	1,101	AACAUGGAACACGAGGAAU
PC-3p-60758_9	4b	19	5,416	853	22,546	8,963	GGAACACGAGGAAUUCUGU
PC-3p-81054_9	4b	20	348	343	17	18	GGACUUGACCAAGAAAUAGA
PC-3p-286752_3	4b	20	748	429	79	50	AAGUCCAAGAGGAAGAGCCA
PC-3p-16688_34	4b	22	515	153	1,007	215	UGAAAAGAACUUUGAAGAGAGA
PC-3p-19200_28	4b	29	718	277	1,867	266	GCGUUUGUGGUAUAGUGGUUAGCAUAGCU
sha-mir-716a-p3_1ss18TC	**3b**	31	523	217	1,272	349	ACGAGAACUUUGAAGGCCGAGGUGGAGAAGG
**miRNA Detected at Low Signals:**							
PC-3p-31854_16	4b	22	8	2	37	10	UCGAGCCCCAGUGGAACCACCA
bta-miR-146b	–	24	12	2	33	6	UGAGAACUGAAUUCCAUAGGCUGU
PC-5p-94376_5	4b	18	86	63	2	3	GCUCUGGAGACCGAGAGU
PC-3p-68123_7	4b	24	36	6	52	6	GUUCAAAUCUCGGUGGAACCUCCA
bta-miR-146a	**1a**	24	22	10	108	63	UGAGAACUGAAUUCCAUAGGUUGU
PC-3p-80933_6	4b	32	17	2	53	21	GGAGACCGGGGUUCAAUUCCCCGACGGGGAGC
PC-3p-117265_4	4b	19	20	26	0	0	AAUGAAUGUAGGGUAUGCU
PC-5p-78389_6	**4a**	19	31	8	1	1	UCAAGUUAUUAAGGGUGUA
PC-5p-13792_44	4b	19	19	32	0	0	AUAUUCCUAGAGAACCCAU
mmu-miR-5097_L+6_1ss23AG	**3a**	30	16	4	34	3	UCCAGGGUUCAUGUCCCUGUUCGGGCGCCA
PC-5p-104508_5	4b	20	43	14	92	18	CGAGCGCCGUUCCGAAGGGA

- :indicates this miRNA has no sequencing group because it was not detected during sequencing.

A recent *in vitro* study demonstrates the involvement of miR-146 in controlling differentiation of spermatogonia. Undifferentiated spermatogonia had increased levels (180-fold higher) of miR-146 over differentiated and an overexpression of miR-146 led to a decrease of transcripts associated with differentiation. Further, retinoic acid, an initiator of spermatogonia differentiation, down-regulated miR-146; and, while miR-146 overexpression blocked the effects of retinoic acid, inhibition of miR-146 enhanced the effects of retinoic acid. Interestingly, miR-146 (a and b) was up-regulated in our treatment group. The role of miR-146 in regulating spermatogonia differentiation is intriguing and, along with our results, suggests a role for environmental influence on spermatogenesis.

A review of the role of miRNA in stress responses by Leung and Sharp [41] describes several studies which have demonstrated how “miRNA mutant animals appear normal…until subjected to stress,” arguing that the role of many specific miRNA may be to help cells function normally in stressful conditions. This is further illustrated by the role of p53, which responds in stressful conditions and activates the production of several miRNA, including many of the miRNA seen in our sequencing results: miR-34c, −16, −26a, and −143. Furthermore, miR-125b (also highly expressed in our sequencing results) has been shown to regulate p53 in humans [42]. A more recent study demonstrated that prolonged stress in mice altered expression of nine sperm miRNA and resulted in changes in offspring responses to stress [43]. Together, these studies suggest a role for miRNA in stress regulation (and perhaps misregulation) as well as illustrate that environmental factors may influence miRNA expression and subsequent affects in offspring [44].

### Predicted Target analysis

The top ten expressed miRNA were predicted to target a total of 3,725 genes, 880 of which were duplicated across the target lists (i.e., targeted by more than one miRNA). All duplicated genes were subjected to GO enrichment analysis to determine what pathways were significantly represented. A total of 27 gene clusters were significantly represented by this group of genes. The top five gene clusters included: (1) regulation of transcription and DNA binding; (2) positive regulation of gene expression and developmental pathways (including: embryonic organ, skeletal system, cartilage, eye, and ectoderm development, among others); (3) Wnt and Hedgehog signaling pathways, as well as several cancer pathways including basal cell carcinoma, colorectal cancer, and thyroid cancer; (4) ATP and nucleotide binding; several signaling pathways including GnRH, ErbB, Wnt, Toll-like receptor; as well as oocyte related pathways, including meiosis, progesterone-mediated oocyte maturation, and focal adhesion; (5) phosphatase activity.

All predicted gene targets for the highest expressed miRNA (miR-100) were also analyzed for GO enrichment. Results indicated fourteen gene pathway “clusters,” many involving developmental processes (tissue, organ, and system development), as well as cellular processes (nucleotide binding, phosphatase activity, and tyrosine kinase receptor signaling). The top ten gene clusters included: (1) biological regulation; (2) developmental process/multicellular organismal development; (3) nucleotide binding; (4) system and organ development; (5) phosphate metabolic process; (6) anatomical structure morphogenesis; (7) nucleus; (8) phosphatase activity; (9) regulation of cellular metabolic process; (10) tissue development.

While several of these gene pathways are present and necessary for spermatogenesis processes, several of these categories appear more likely to be pertinent to zygote formation and development rather than to sperm production. For instance, four of the top ten gene clusters for miR-100 specifically reference developmental processes (#2, 4, 6, and 10 in the list above). While several of them simply reference general cellular pathways, these pathways could be relevant both in the developing spermatozoa as well as the developing zygote (#1, 3, 5, 7, 8, 9). Furthermore, when looking at the top-ten sequenced miRNA in sperm, the gene cluster pathways also indicate potential roles for sperm miRNA in the developing zygote. For instance, for gene cluster #2, several developmental processes relevant to embryonic development were specifically indicated. Furthermore, wnt and hedgehog pathways are both well known for their prominent roles in embryogenesis.

In conclusion, while fescue toxicosis appeared to have little effect upon sperm miRNA expression (with the possible exception of miRNA-146a), the miRNA profile of mature ejaculated sperm may in fact have downstream consequences upon embryonic development. The potential for sperm miRNA affecting zygote development has recently been reported in the literature [18] and has interesting implications for the use of sperm miRNA profiles as indicators of potential male fertility.
